# Advanced HIV disease at presentation to care in Nairobi, Kenya: late diagnosis or delayed linkage to care?—a cross-sectional study

**DOI:** 10.1186/s12879-016-1500-8

**Published:** 2016-04-18

**Authors:** Mia Liisa van der Kop, Lehana Thabane, Patricia Opondo Awiti, Samuel Muhula, Lennie Bazira Kyomuhangi, Richard Todd Lester, Anna Mia Ekström

**Affiliations:** Department of Public Health Sciences/Global Health (IHCAR), Karolinska Institutet, Widerströmska Huset, Tomtebodavägen 18A, Stockholm, 171-77 Sweden; Department of Medicine, University of British Columbia, 828 West 10th Avenue, Vancouver, BC V5Z 1M9 Canada; Department of Clinical Epidemiology and Biostatistics, McMaster University, 50 Charlton Avenue East, Hamilton, ON L8N 4A6 Canada; Amref Health Africa, Langata Road, Nairobi, Kenya; Department of Infectious Diseases, I73, Karolinska University Hospital, 141 86 Stockholm, Sweden

**Keywords:** HIV/AIDS, Sub-Saharan Africa, Kenya, Advanced HIV, Presentation to HIV care, Informal settlements

## Abstract

**Background:**

Presenting to care with advanced HIV is common in sub-Saharan Africa and increases the risk of severe disease and death; however, it remains unclear whether this is a consequence of late diagnosis or a delay in seeking care after diagnosis. The objectives of this cross-sectional study were to determine factors associated with advanced HIV at presentation to care and whether this was due to late diagnosis or delays in accessing care.

**Methods:**

Between 2013 and 2015, adults presenting to care were recruited at two clinics in low-income areas of Nairobi, Kenya. Participants were considered to have advanced HIV if their CD4 count was below 200 cells/μL, or they were in WHO stage 4. Information on previous HIV diagnoses was collected using interviewer-administered questionnaires. Logistic regression was used to determine the association between clinical and socio-demographic factors and advanced HIV.

**Results:**

Of 753 participants presenting to HIV care, 248 (33 %) had advanced HIV. Almost 60 % (146/248) of those presenting with advanced HIV had been previously diagnosed, most of whom (102/145; 70 %) presented to care within three months of their initial diagnosis. The median time to presentation to HIV care after an initial diagnosis was 22 days (IQR 6-147) for those with advanced HIV, compared to 19 days (IQR 4-119) for those with non-advanced HIV (*p* = 0.716). Clinic (adjusted odds ratio [AOR] 1.55, 95 % CI 1.09–2.20) and age (AOR 1.72 per unit increase in age category, 95 % CI 1.45–2.03) were associated with presenting with advanced HIV.

**Conclusions:**

Presentation to care with advanced HIV was primarily due to delayed diagnosis, rather than delayed linkage to care after diagnosis. Variation by clinic suggests that outreach and other community-based efforts may drive earlier testing and linkage to care. Our findings highlight the ongoing importance of implementing strategies to encourage earlier HIV diagnosis, particularly among adults 30 years and older.

## Background

### Rationale

Despite increased HIV testing, improved global access to antiretroviral therapy (ART), and changes in World Health Organization (WHO) recommendations to initiate treatment earlier, the problem of presenting to care with low CD4 counts persists across sub-Saharan Africa. Those who present to care with a CD4 count below 200 cells/μL, or an AIDS-defining event, are considered to have advanced HIV disease [1]. Individuals who initiate care with advanced HIV are more likely to have impaired immune recovery [2] and reduced life expectancy [3]. Presenting with advanced HIV may also result in higher direct medical and societal costs [4, 5], as well as an increased risk of onward transmission [6]. Furthermore, high proportions of individuals presenting with advanced HIV will impede the UNAIDS 90-90-90 targets to have 90 % of those living with HIV aware of their status; 90 % of those diagnosed on treatment; and 90 % of those on treatment virologically suppressed [7]. Understanding risk factors for presentation to care with advanced HIV is critical to developing strategies to encourage earlier linkage to care and successful therapy.

While sub-Saharan Africa is disproportionately affected by the HIV epidemic and persons present to care with significantly lower CD4 counts than in other settings [8, 9]; the majority of research on presentation to care with advanced HIV has been conducted in higher-resource settings. Of studies in sub-Saharan Africa, several relied on routine clinical data, and although these studies contributed important insights into presentation to care with advanced HIV disease, they were unable to capture data on important variables and suffered from large amounts of missing data [10–12]. Other studies have been limited by small sample size [12, 13], and there have been contradictory findings on some of the important determinants of presenting with advanced HIV, for example, age and alcohol use [9, 11, 12, 14, 15]. Furthermore, previous research in the region lacked data on timing of HIV diagnosis. There has been a strong call to determine whether it is a delay in diagnosis or a delay in seeking care *after* diagnosis that leads to presentation to care with advanced HIV [9, 10, 16, 17]. Here, we conduct a cross-sectional study of persons presenting at two clinics in low-income areas of Nairobi, Kenya to evaluate the pathway to presentation to care with advanced HIV disease and its associated factors.

### Objectives

1. Quantify the proportion of individuals who first present to care with advanced HIV.
2. Determine whether presenting to care with advanced HIV was due to delayed diagnosis or a delay in seeking care after diagnosis.
3. Determine factors associated with first presentation to care with advanced HIV.

## Methods

### Study design

This cross-sectional study used baseline data collected during a randomized controlled trial and supplementary cohort study. The trial involves evaluating the effectiveness of a text-messaging intervention to improve retention in early HIV care [18]. Patients who did not fulfil phone-related eligibility criteria for the trial were invited to participate in a supplementary cohort study to examine patient retention during the first year of HIV care. Adults testing positive for HIV at two comprehensive care clinics in Nairobi, Kenya were assessed for study eligibility.

### Study setting and participants

Between April 2013 and June 2015, participants were recruited from the Kibera Community Health Centre, an Amref Health Africa clinic located in a large informal settlement. At this comprehensive care clinic, there are no direct patient costs for HIV care and treatment. The population the clinic serves lacks or has minimal access to services such as education, water, sanitation, or other public services. HIV prevalence among adults tested for the first time is estimated at 13 % [19]. In March 2014, recruitment began at a second comprehensive care clinic, the Baba Dogo Health Centre, which is situated in another large informal settlement in Nairobi’s Eastlands area and operated by the Kenya AIDS Control Project.

At each clinic, clinical staff introduced potential participants to a research nurse, who completed an eligibility assessment. Patients were eligible to participate in the study if they were 18 years old or older, HIV-positive, and willing to provide informed consent. Patients previously assessed for ART eligibility, with prior ART exposure, or on ART were excluded. Women known to be pregnant were also excluded. Patients were screened for study participation at the time of a positive HIV diagnosis, although potential participants had one week to decide whether to enroll in the study. Screened patients were a mixture of those who: 1) presented to the clinic for HIV testing and counselling (HTC) services; 2) sought treatment for an illness and then the clinician referred them to HTC; 3) had been diagnosed with HIV elsewhere and presented to the clinic specifically to receive HIV care.

### Outcomes

#### Presentation with advanced HIV disease

Presentation with advanced HIV disease was defined as presenting with a CD4 count <200 cells/μL or at WHO stage 4, regardless of CD4 count. This definition is based on the consensus definition of advanced HIV disease, which is presenting with a CD4 count <200 cells/μL or an AIDS-defining event, regardless of CD4 count [1]. WHO Stage 4 was used as a proxy for AIDS-defining events because of the overlap between AIDS-defining conditions and the clinical events comprising WHO stage 4 [20]. Presentation to care was defined as “attendance at a health care facility that is able to monitor progression of HIV infection and initiate appropriate medical care, including ART, as appropriate” [1].

#### Delay in seeking care

A delay in seeking care was defined as presenting to care more than three months after a previous HIV diagnosis.

#### Determinants of presenting with advanced HIV disease and potential effect modifiers

Variables were selected if there was prior strong evidence of their association with advanced HIV disease at presentation, e.g., sex (male or female) [9, 10, 14] and education (some secondary versus no secondary) [9, 12, 14]; or if evidence was conflicting, e.g., age (<30; 30–39; 40–49; ≥60 years) [9, 14, 21], travel time to the clinic (<30, 30–59, ≥60 min) [9, 14], and alcohol use (hazardous drinking versus non- hazardous drinking, [9, 12, 15] identified by the AUDIT-C questionnaire score) [22]. We also investigated a novel individual-level variable that may be a factor in presenting with advanced HIV, current illicit drug use (within 30 days of the baseline visit) e.g., heroin, cocaine, etc. Since clinics are operated by different organizations and serve different populations, clinic attended was also considered (Baba Dogo v. Kibera). *A priori* information was not available on potential interaction between factors associated with presentation to care with advanced HIV; therefore, we explored plausible interaction between sex and travel to time to clinic, as an interactive effect has been found in studies investigating retention in HIV care [23, 24].

### Data sources and measurement

At the baseline visit, the research nurse administered a questionnaire in the participant’s language of choice, English or Kiswahili. Prior to starting recruitment, the questionnaire was translated from English to Kiswahili, back-translated, and pre-tested with clinic patients (*n* = 10). The questionnaire collected information on demographic characteristics, HIV testing history, and substance use. Blood was drawn at the baseline visit for laboratory CD4 testing. HIV and CD4 testing were consistent with routine clinical practice. Data were entered in Microsoft Access on a weekly basis. Verification procedures included cross-checking data files with original forms and clinical records, as well as range and consistency checks.

### Study size

A conservative rule is that logistic regression models should have 10 outcome events per predictor variable to build stable models [25]. A preliminary descriptive analysis indicated that there were 152 events of presentation to care with advanced HIV in this cohort, [26] which was adequate to build stable models with the six selected factors.

### Statistical methods

Descriptive analyses of the study population, including the proportion of patients presenting with advanced HIV disease, were conducted in SPSS v14. To compare the time to presentation to care between advanced HIV and non-advanced HIV groups (for those with a previous diagnosis), a Mann–Whitney U test was used. Analyses were restricted to individuals with complete data.

Logistic regression was used to determine factors associated with advanced HIV at presentation to care. First, univariable analyses were performed to assess the strength of the association between each factor and the outcome. Variables were then included in an initial multivariable model if they had a univariable *p*-value of ≤0.25 or were considered important based on prior evidence (i.e., sex). In the final adjusted models, variables were selected based on a significance threshold of *p* < 0.05. Nested models were compared using likelihood ratio tests to examine interaction between sex and travel time, and to determine whether to include a linear effect or indicator variables for ordered categorical variables. The fit of the final model was tested with the Hosmer-Lemeshow goodness-of-fit test [27]. Results are presented as estimated odds ratios (OR) and adjusted ORs (AOR) with corresponding 95 % confidence intervals (CI) and *p*-values. All *p*-values are two-sided and reported to three decimal places with those less than 0.001 reported as *p* < 0.001. Analyses were performed using Stata version 12 (Statacorp, College Station, TX).

### Ethics

The study protocol was approved by the University of British Columbia’s Clinical Research Ethics Board (H12-00563) and Amref Health Africa’s Ethics and Scientific Review Committee (P40/12).

## Results

### Study population

**Fig. 1:**
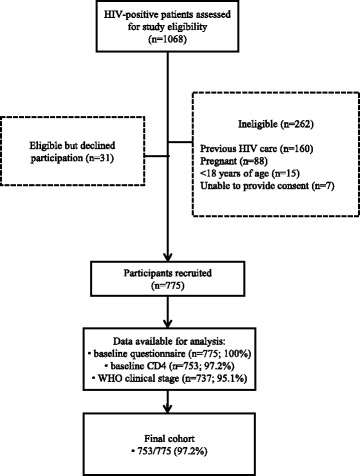
Participant recruitment flow diagram

Between April 2013 and June 2015, 1068 HIV-positive individuals presenting to the Baba Dogo and Kibera Health Centres were screened for study participation, and 775 were recruited (Fig. 1). The most frequent reasons patients were ineligible to participate were previous enrolment in HIV care (*n* = 160/262; 61 %) and pregnancy (*n* = 88/262; 34 %). Less than 3 % of screened participants (*n* = 31/1068) declined participation. Of the 775 participants recruited, baseline CD4 data were available for 97.2 % (*n* = 753/775) of the cohort.

The mean age of participants was 34 years (standard deviation 9.82), and males comprised 40 % of the cohort. Additional demographic and clinical characteristics are summarized in Table 1. The median baseline CD4 count was 302 cells/μL (IQR 148-463); 60.7 % (457/753) had a CD4 count lower than 350 cells/μL. Approximately 1/3 (*n* = 248/753; 32.9 %) of the cohort presented to care with advanced HIV (CD4 count <200 cells/μL or WHO stage 4).

**Table 1:** Demographic and clinical characteristics of participants. Values are numbers (percentages)

Variable	Non-advanced HIV at presentation to care (*n* = 505)	Advanced HIV at presentation to care (*n* = 248)
Sex		
Male	183 (61.4)	115 (38.6)
Female	322 (70.8)	133 (29.2)
Age (years)		
Mean (SD)	32 (9.22)	37 (10.26)
<30	227 (79.9)	57 (20.1)
30–39	178 (62.9)	105 (37.1)
40–49	71 (57.7)	52 (42.3)
≥50	29 (46.0)	34 (54.0)
Education		
No secondary school	327 (64.9)	177 (35.1)
Some secondary school	178 (71.5)	71 (28.5)
Clinic		
Kibera	364 (68.4)	168 (31.6)
Baba Dogo	141 (63.8)	80 (36.2)
CD4		
Median (IQR) (cells/μL)	389 (298–545)	90 (42–147)
≤350	210 (46.0)	247 (54.0)
>350	295 (99.7)	1 (0.3)
WHO Stage		
1	356 (78.1)	100 (21.9)
2	72 (63.7)	41 (36.3)
3	57 (39.9)	86 (60.1)
4	0 (0.0)	8 (100.0)
Missing	20 (60.6)	13 (39.4)
Previous HIV diagnosis		
No	199 (66.1)	102 (33.9)
Yes	306 (67.7)	146 (32.3)
Travel time to clinic		
<30 min	236 (63.6)	118 (36.4)
30–59 min	219 (70.9)	90 (29.1)
≥60 min	77 (67.5)	37 (32.5)
Missing	3 (50.0)	3 (50.0)
Alcohol use		
Non-heavy/hazardous drinking	356 (67.3)	173 (32.7)
Heavy/hazardous drinking	149 (66.5)	75 (33.5)
Illicit drug use		
Not a current drug user	469 (66.4)	237 (33.6)
Current drug user	36 (76.6)	11 (23.4)

*Abbreviations: SD* standard deviation, *IQR* interquartile range

### Late diagnosis versus delayed presentation to care

Of those who presented to care with advanced HIV, 146 (59 %) had been previously diagnosed with HIV. This was similar to the proportion of those with a previous diagnosis in the non-advanced HIV group (*n* = 306/505; 61 %; chi-square *p*-value 0.650). Most participants with advanced HIV presented to care within three months of their initial diagnosis (102/145; 70 %), including 44 individuals who presented within one week. Data on the date of first HIV diagnosis was missing for one participant. The median time to presentation to HIV care after an initial diagnosis was 22 days (IQR 6-147) for those with advanced HIV, compared to 19 days (IQR 4-119) for those with non-advanced HIV (*p* = 0.716).

### Factors associated with presentation to care with advanced HIV

Table 2 shows the association between clinical and sociodemographic characteristics and presenting to care with advanced HIV. In both univariable and multivariable analyses, age was linearly associated with presenting to care with advanced HIV, with a final AOR of 1.72 (95 % CI 1.45 to 2.03) per unit increase in age category, compared to the reference category of <30 years. Individuals presenting to the Baba Dogo clinic were more likely to present with advanced HIV (AOR 1.55; 95 % CI 1.09–2.20) than those at the Kibera clinic. Those with some secondary education were less likely to present with advanced HIV; however this association was of borderline significance in the final model (AOR 0.73; 95 % CI 0.53–1.03). In the univariable analysis, male sex appeared to be associated with presenting with advanced HIV; however, this effect diminished in the multivariable analysis and did not remain in the final model.

**Table 2:** Univariable and multivariable analysis of variables associated with presentation to care with advanced HIV disease

	Crude ORs			Adjusted ORs			Final adjusted ORs		
Variable	OR	95 % CI	*p*-value	OR	95 % CI	*p*-value	OR	95 % CI	*p*-value
Age^a^	1.66	1.41–1.96	<0.001	1.65	1.39–1.97	<0.001	1.72	1.45–2.03	<0.001
Presenting at the Baba Dogo clinic	1.23	0.88–1.71	0.220	1.53	1.08–2.17	0.018	1.55	1.09–2.20	0.014
Secondary education	0.74	0.53–1.03	0.070	0.69	0.49–0.98	0.040	0.73	0.52–1.03	0.073
Male gender	1.52	1.12–2.07	0.008	1.30	0.93–1.82	0.128			
Illicit drug use	0.60	0.30–1.20	0.155	0.53	0.26–1.09	0.084			
Hazardous drinking	1.04	0.74–1.44	0.835						
Travel time^b^	0.98	0.84–1.34	0.782						
Previous HIV diagnosis	0.93	0.68–1.27	0.650						

*Abbreviations: OR* odds ratio, *CI* confidence interval

^a^OR corresponds to an increase in the odds ratio per unit increase in age category (<30 years, 30–39 years, 40–49 years, ≥50 years)

^b^OR corresponds to an increase in the odds ratio per unit increase in travel time category (<30 min, 30–59 min, ≥60 min)

Hosmer-Lemeshow goodness-of-fit *p* = 0.199

## Discussion

### Key results

In this cohort of individuals presenting to HIV care at two clinics in Nairobi, Kenya, approximately one-third presented to care with advanced HIV, suggesting important opportunities still exist to encourage earlier diagnosis and treatment. We found that delayed diagnosis was more common than delayed linkage to care in explaining presentation to care with advanced HIV. Although 59 % of those presenting with advanced HIV had been previously diagnosed with HIV, almost ¾ of these individuals presented to care within three months of their initial diagnosis. Given the average rate of decline of CD4 T lymphocytes [28, 29], it is unlikely that many of the individuals who presented to care with advanced HIV within three months of their previous diagnosis would have had non-advanced HIV when they were initially diagnosed.

Overall, there was a strong linear increase in the likelihood of presenting with advanced HIV among age groups older than 30. The proportion of those presenting with advanced HIV also varied by clinic, but this may be expected as larger structural and contextual correlates are likely to vary in different care settings. In this instance, we noted that Baba Dogo lacks the same level of community outreach programs present in Kibera. Community outreach programs in Kibera include home-based HIV testing and counselling (HBTC) by various organizations, including Amref Health Africa, Liverpool VCT, and the Centers for Disease Control and Prevention (CDC). Kibera also benefits from numerous clinics at which HIV can be tested, including a Médicins Sans Frontières (MSF) clinic that opened during the time of recruitment for this study. While HBTC efforts exist in Baba Dogo, they are less prevalent. These community-based efforts may drive earlier testing and linkage to care, and may have led to comparatively fewer clients presenting with advanced HIV in Kibera.

### Comparability with other studies

Various definitions of ‘advanced HIV disease’ have been used in the literature, and the term has frequently been used interchangeably with the term ‘late presentation’. Definitions of ‘advanced HIV disease’ from studies in sub-Saharan Africa have included: CD4 count <100 cells/μL or WHO stage 4 [10]; WHO stage 3 or 4 [9]; and CD4 < 100 cells/μL [14]. The wide array of definitions used makes it difficult to compare the proportions of individuals presenting to care with advanced HIV across studies. For instance, in a large, multi-country study by Lahuerta et al., 19 % of those enrolling in care were classified as having advanced HIV [10], compared to 33 % in our study; however, Lahuerta et al. used a lower CD4 threshold of 100 cells/μL, so more individuals might have been classified as having advanced HIV than if a higher threshold of 200 cells/μL had been used. The recent development of consensus definitions of ‘late presentation’ (CD4 below 350 cells/μL or presenting with an AIDS-defining event), ‘presentation with advanced HIV’, and even ‘presentation for care’ [1, 17], and their use going forward, will facilitate comparison between studies in the future.

During the course of this study, the clinics transitioned from initiating treatment at CD4 counts of 350 cells/μL or less to the 2013 WHO’s recommendations to initiate treatment at 500 cells/μL or lower [30]. The benefits of which include improved survival, immune recovery, and a decreased risk of transmission [30]. With approximately one-third of patients presenting to care with advanced HIV, and over half of the population presenting with a CD4 count lower than 350 cells/μL, the majority of patients at these clinics will not be affected by the change in treatment guidelines, or more recent recommendations to initiate treatment upon diagnosis, regardless of CD4 count. Over the past decade, CD4 count at presentation has not markedly increased in sub-Saharan Africa [7], and while it is too early to tell whether implementation of the new guidelines will promote earlier presentation to care, our study emphasizes that it is critical to develop and implement strategies that encourage earlier diagnosis. Without this, stated targets of expansion of therapy to those who are eligible and the intended individual- and population-level effects of the new WHO recommendations will not be fully realised.

The importance of earlier diagnosis is further supported by our findings that presentation with advanced HIV was largely due to delayed diagnosis, rather than a delay in seeking care after diagnosis. Prior studies on presentation with advanced HIV in the region did not examine prior diagnoses [13], considered new diagnoses only [14], or were based on clinical records [9, 10], thereby restricting their ability to investigate the pathway to presentation with advanced HIV. In addition to our finding that approximately ¾ of those with advanced HIV who had been previously diagnosed presented to care within three months, the proportion of individuals who had had a previous diagnosis was similar between those with or without advanced HIV, and the median time to first presentation to HIV care did not significantly differ between the two groups. This supports our conclusion that advanced HIV at first presentation was primarily due to delayed diagnosis. This is not to underestimate the importance of promoting timely linkage to care; however, as almost ¼ of those with advanced HIV (who had been previously diagnosed) took longer than three months to present to care, and many individuals who test positive do not link to care [31, 32].

Similar to Kigozi et al. in their Ugandan study [9], we found that older age was associated with advanced HIV at presentation. This may be due to simply having lived long enough for the disease to progress to an advanced stage, or age-associated differences in HIV awareness, knowledge or stigma that may affect testing and other care-seeking behaviours [33]. Reducing the barriers to and encouraging earlier diagnosis among older adults is particularly important because of the smaller gains made in CD4 response and increased risk of mortality compared to younger age groups once ART is initiated [33-35]. A study from South Africa found no association with age and presentation with advanced HIV [15]; however, in the South African study, age was dichotomized at 40 years, which may have underestimated the variation in risk according to age [36].

Although not statistically significant, relatively more men than women presented to care with advanced HIV. This is in contrast to other reports [9, 10, 14], which found strong associations between male gender and presentation with advanced disease. This may have been due to the exclusion of pregnant women from this study. In studies on CD4 at presentation to care, those with a focus on prevention of mother-to-child transmission (PMTCT) reported a higher mean CD4 count at presentation (395 cells/μL) [7] than non-PMTCT-focussed studies, and those enrolling in PMTCT services have been found to have a lower likelihood of presenting with advanced HIV disease than others [10]. By excluding pregnant women in this study, the difference between the genders in the risk of presenting with advanced HIV may have been attenuated.

Other factors of interest, such as educational level [9, 12], hazardous drinking [9, 12, 15], and travel time to clinic [9], have been found to be associated with presentation to care with advanced HIV in previous studies but were not in our cohort. There are several possible explanations for this beyond the different populations under study. First, the use of varying definitions of advanced HIV may underlie the differing results: factors that are associated with advanced HIV when a lower CD4 threshold is used may not be similarly associated with advanced HIV when the consensus definition is applied. Other possible explanations include high levels of missing data in some previous studies, which may have impacted findings; investigation of a large number of variables, increasing the likelihood of chance findings; and a larger sample size in some of the previous studies, increasing the power to detect effects.

### Strengths and limitations of the study

One of this study’s principal strengths is our collection of information on the date of participants’ first HIV diagnosis, helping us to illuminate the pathway to presentation to care with advanced HIV. A second major strength of this study is the completeness of the data: CD4 data was available for 97 % of the cohort, and information on additional variables, such as alcohol or illicit drug use, was available for all participants. Additional strengths include a high participation rate, which minimized the possibility of non-participation bias. This, combined with the inclusion of two sites in the study, improves this study’s generalizability.

We used a consensus definition of advanced HIV, part of which is based upon presenting with an AIDS-defining event regardless of CD4 count. WHO stage 4 was used as a proxy for AIDS-defining events. Although most AIDS-defining conditions are included in WHO stage 4, WHO staging data was only available for 85 % of the cohort, which may have led to the misclassification of some participants as non-advanced HIV. Furthermore, CD4 was measured at only one point in time. Given laboratory variability in CD4 measurements, and the possibility that other factors may temporarily influence CD4 counts [1], it would have been preferable to have a confirmatory CD4 count.

Other limitations include the self-reported nature of data on the occurrence and timing of prior HIV diagnoses. There is a lack of data from Kenya on the validity of self-reported HIV testing data; however, studies from other parts of sub-Saharan Africa suggest that HIV-positive individuals may underreport past testing [37, 38]. While the reasons for underreporting a previous diagnosis are not well understood, one possibility is that individuals fear being turned away from the clinic if they indicate they are aware of their status. At the study sites, care and treatment guidelines were developed to protect clients from being denied care. It is standard clinical practice to test all patients who come to the clinics for HIV care, regardless of whether they have been tested or diagnosed with HIV before. These guidelines may encourage more honest reporting on previous diagnoses than might otherwise occur. To improve the quality of self-reported data, participants completed the questionnaire after being assured of confidentiality. In addition, the questionnaire was administered by an experienced HIV research nurse in a private room. Finally, data from this section of the questionnaire were cross-checked with a later section to assess consistency. A high degree of consistency was found, and any discrepancies were investigated further and resolved. Despite the limitations inherent in self-reported data, the study is unlikely to suffer from recall bias. There is no strong reason to believe that the advanced HIV versus non-advanced HIV groups would differentially report events; however, data on the dates of previous HIV diagnoses may be less valid than if we had had access to clinical records.

## Conclusions

Presentation to care with advanced HIV continues to burden global HIV programs. In our study, this appeared to be largely due to delayed diagnosis, rather than delays in seeking care after diagnosis. The benefits of early HIV care and treatment for both individual health reasons, and population benefits through treatment as prevention, are now widely accepted. Efforts are needed to maximize earlier diagnosis and entry into care at the front end of the HIV care continuum to fulfil new global targets. Otherwise, changing guidelines to recommend treatment earlier in the course of HIV infection will not achieve their intended outcomes.

## Ethics approval and consent to participate

The study protocol was approved by the University of British Columbia’s Clinical Research Ethics Board (H12-00563) and Amref Health Africa’s Ethics and Scientific Review Committee (P40/12). Individuals provided written informed consent to participate.

## Consent for publication

Not applicable.

## Availability of data and materials

Data supporting our findings will be shared upon request.

## Abbreviations

AIDS: Acquired Immune Deficieny Syndrome
AOR: adjusted odds ratio
ART: antiretroviral therapy
AUDIT-C: Alcohol Use Disorders Identification Test
CD4: cluster of differentiation 4
CI: confidence interval
HIV: Human Immunodeficiency Virus
HTC: HIV testing and counselling
IQR: interquartile range
OR: odds ratio
SPSS: Statistical Package for the Social Sciences
TX: Texas
WHO: World Health Organization
